# Supplementary material for: Development, system design, safety, and performance metrics of a conversational agent for reducing depressive and anxious symptoms based on a large language model: The MHAI study
Source: PLoS One. 2026 Mar 18;21(3):e0344939. doi: 10.1371/journal.pone.0344939 (PMC12998858; doi:10.1371/journal.pone.0344939)
Supplement: S1 File — (DOCX) [file pone.0344939.s001.docx]

**S1 File.** CHART checklist.

| **Heading** | **#** | **CHART checklist item** | **Page #*** |
| --- | --- | --- | --- |
| **Title & abstract** |  |  |  |
| Title | **1a** | State that the study is assessing one or more generative AI-driven chatbots for clinical evidence or health advice. | pp1 |
| Abstract/Summary | **1b** | Apply a structured format, if applicable. | pp3 |
| **Introduction** |  |  |  |
| Background | **2a** | State the scientific background, rationale, and healthcare context for evaluating the generative AI-driven chatbot(s), referencing relevant literature when applicable. | pp4 |
|  | **2b** | State the aims and research questions including the target audience, intervention, comparator(s), and outcome(s). | pp4 |
| **Methods** |  |  |  |
| Model identifiers | **3a** | State the name and version identifier(s) of the generative AI model(s) and chatbot(s) under evaluation, as well as their date of release or last update. | pp5 |
|  | **3b** | State whether the generative AI model(s) and chatbot(s) are open-source or closed-source/proprietary. | pp5 |
| Model details | **4a** | State whether the generative AI model was a base model or a novel base model, tuned model, or fine-tuned model. | pp5 |
|  | **4b** | If a base model is used, cite its development in sufficient detail to identify the model. | pp5 |
|  | **4c** | If a novel base model, tuned model, or fine-tuned model is used, describe the pre- and/or post-implementation/deployment data and parameters. | NA |
| Prompt engineering | **5a** | Describe the evolution of study prompt development. | pp5, pp18 |
|  | **5ai** | Describe the sources of prompts. | pp5, pp18 |
|  | **5aii** | State the number and characteristics of the individual(s) involved in prompt engineering. | pp18 |
|  | **5aiii** | Provide details of any patient and public involvement during prompt engineering. | pp18 |
|  | **5b** | Provide study prompts. | pp18 |
| Query strategy | **6a** | State route of access to generative AI model. | pp5 |
|  | **6b** | State the date(s) and location(s) of queries for the generative AI-driven chatbot(s) including the day, month, and year as well as city and country. | pp5 |
|  | **6c** | Describe whether prompts were input into separate chat session(s). | NA |
|  | **6d** | Provide all generative AI-driven chatbot output/responses | pp3 |
| Performance evaluation | **7a** | Define the ground truth or reference standard used to define successful generative AI-driven chatbot performance. | NA |
|  | **7b** | Describe the process undertaken for generative AI-driven chatbot performance evaluation. | pp7 |
|  | **7bi** | State the number and characteristics of team members involved in performance evaluation. | pp6 |
|  | **7bii** | Provide details of any patients and public involvement during the evaluation process. | NA |
|  | **7biii** | State whether evaluators were blinded to the identity of the generative AI-driven chatbot(s) under assessment. | NA |
| Sample size | **8** | Report how the sample size was determined. | pp6 |
| Data analysis | **9a** | Describe statistical analysis methods, including any evaluation of reproducibility of generative AI-driven chatbot responses. | pp8 |
|  | **9ai** | Report the measures used for performance evaluation. | pp8, pp10 |
| **Results** | **10a** | Report the performance evaluation undertaken including the alignment between generative AI-driven chatbot output and ground truth or reference standard using quantitative or mixed methods approaches as applicable. | Pp9-11 |
|  | **10b** | For responses deviating from the ground truth or reference standard, state the nature of the difference(s). | pp10-11 |
|  | **10c** | Report the evaluation for potentially harmful, biased, or misleading responses. | pp10 |
| **Discussion** | **11a** | Interpret study findings in the context of relevant evidence. |  |
|  | **11b** | Describe the strengths and limitations of the study. |  |
|  | **11c** | Describe the potential implications for practice, education, policy, regulation, and research. |  |
| **Open science** |  |  |  |
| Disclosures | **12a** | Report any relevant conflicts of interest for all authors. | pp2 |
| Funding | **12b** | Report sources of funding and their role in the conduct and reporting of the study. | pp2 |
| Ethics | **12c** | Describe the process undertaken for ethical approval. | pp2 |
|  | **12ci** | Describe the measures taken to safeguard data privacy of patient health information, as applicable. | NA |
|  | **12cii** | State whether permission/licensing was obtained for the use of original, copyrighted data. | NA |
| Protocol | **12d** | Provide a study protocol. | NA |
| Data availability | **12e** | State where study data, code repository, and model parameters can be accessed. | pp2 |
