# Supplementary material for: Development, system design, safety, and performance metrics of a conversational agent for reducing depressive and anxious symptoms based on a large language model: The MHAI study
Source: PLoS One. 2026 Mar 18;21(3):e0344939. doi: 10.1371/journal.pone.0344939 (PMC12998858; doi:10.1371/journal.pone.0344939)
Supplement: S3 File — (DOCX) [file pone.0344939.s003.docx]

**S3 File.** User personas.

User Persona 1: Ruby (Depression)

- Age: 52
- Gender: Female
- Occupation: High school teacher
- Marital status: Divorced
- Context: Lives alone since her children moved out. She has been feeling low on energy, unmotivated, and has lost interest in teaching, which she used to enjoy. She struggles with sleep and has lost weight without trying.
- Reason for seeking help: She wants to understand why she has been feeling so sad and empty for several months. She sometimes feels like her life has no purpose.

User Persona 2: Mahony (Anxiety)

- Age: 28
- Gender: Male
- Occupation: Financial analyst
- Marital status: Single
- Context: Lives in a city far from his family. Has been experiencing palpitations, shortness of breath, and catastrophic thoughts before presenting at work. Finds it hard to focus and is constantly afraid of making mistakes. He avoids meetings due to fear of being judged.
- Reason for seeking help: He wants to manage his anxiety, as it's starting to affect his performance at work and personal relationships.

User Persona 3: Amy (Depression + Anxiety)

- Age: 19
- Gender: Female
- Occupation: First-year university student
- Marital status: Single
- Context: Lives in a university dorm far from home. Frequently cries without clear reason, feels empty, and has a constant fear of failure. She has stopped attending classes and avoids hanging out with friends. She suffers from insomnia and has recurring negative thoughts.
- Reason for seeking help: She wants to feel like herself again, regain motivation, and stop feeling stuck between sadness and constant worry.
