# Supplementary material for: Development, system design, safety, and performance metrics of a conversational agent for reducing depressive and anxious symptoms based on a large language model: The MHAI study
Source: PLoS One. 2026 Mar 18;21(3):e0344939. doi: 10.1371/journal.pone.0344939 (PMC12998858; doi:10.1371/journal.pone.0344939)

**S4 File.** APP Workflow.

1. Registration and login page.
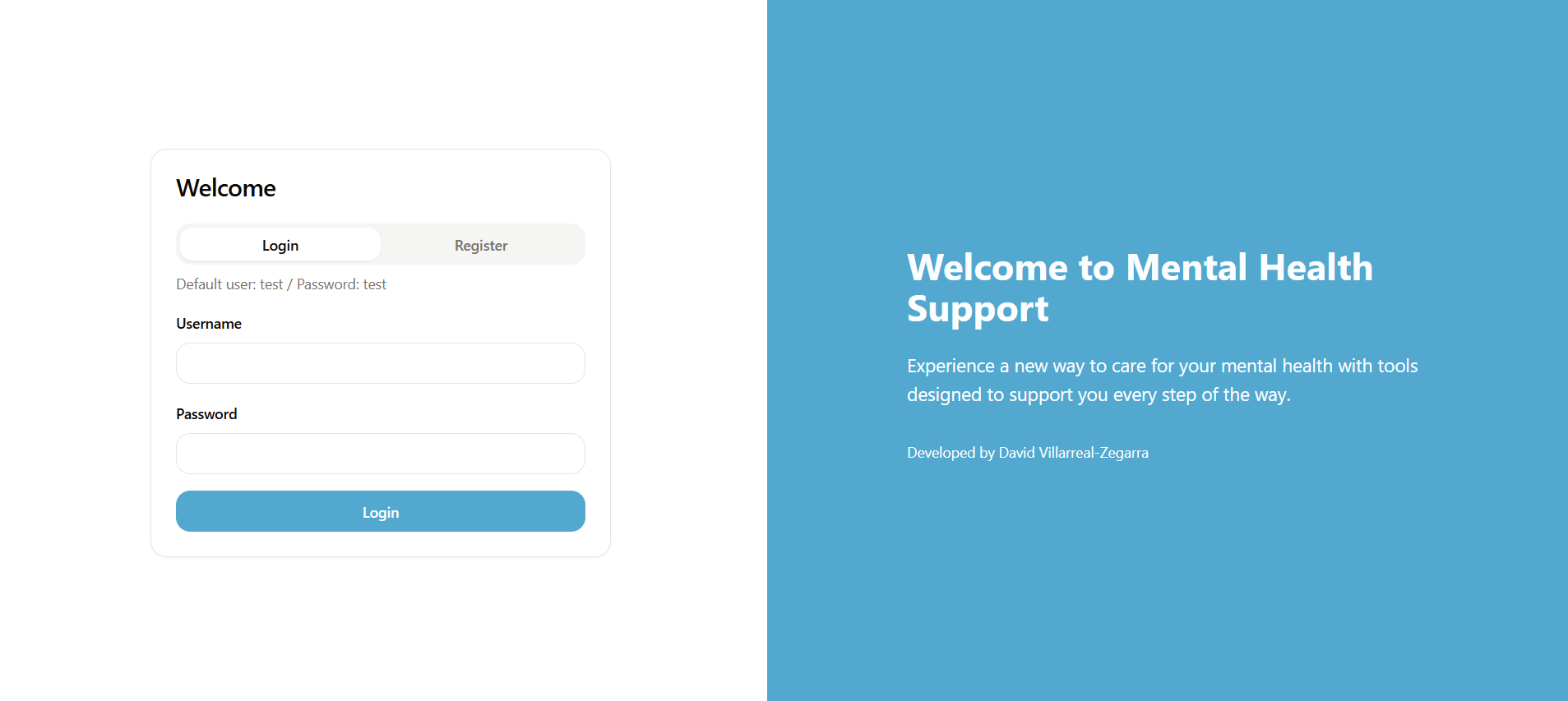


2. APP dashboard.


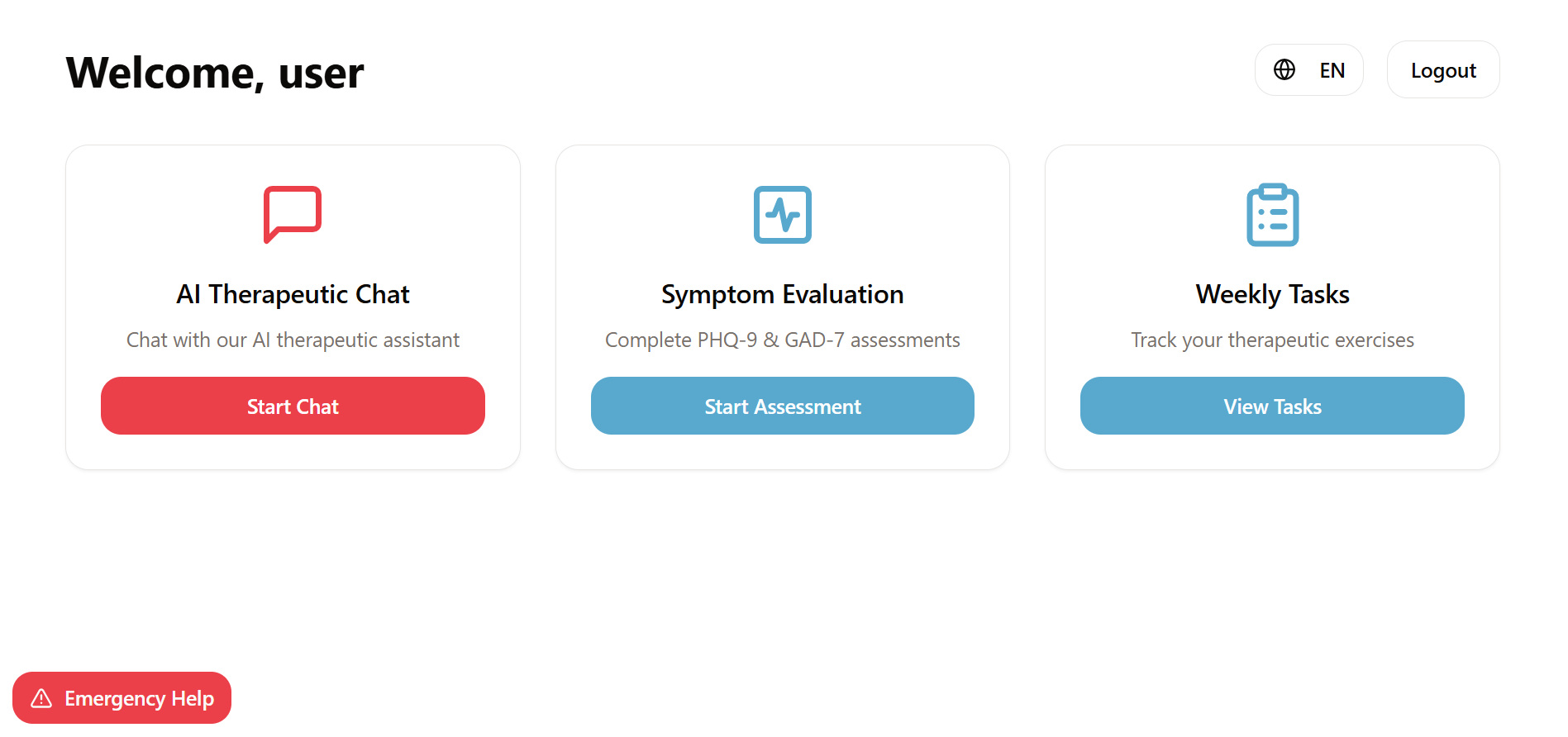


3. Therapeutic chat with AI.


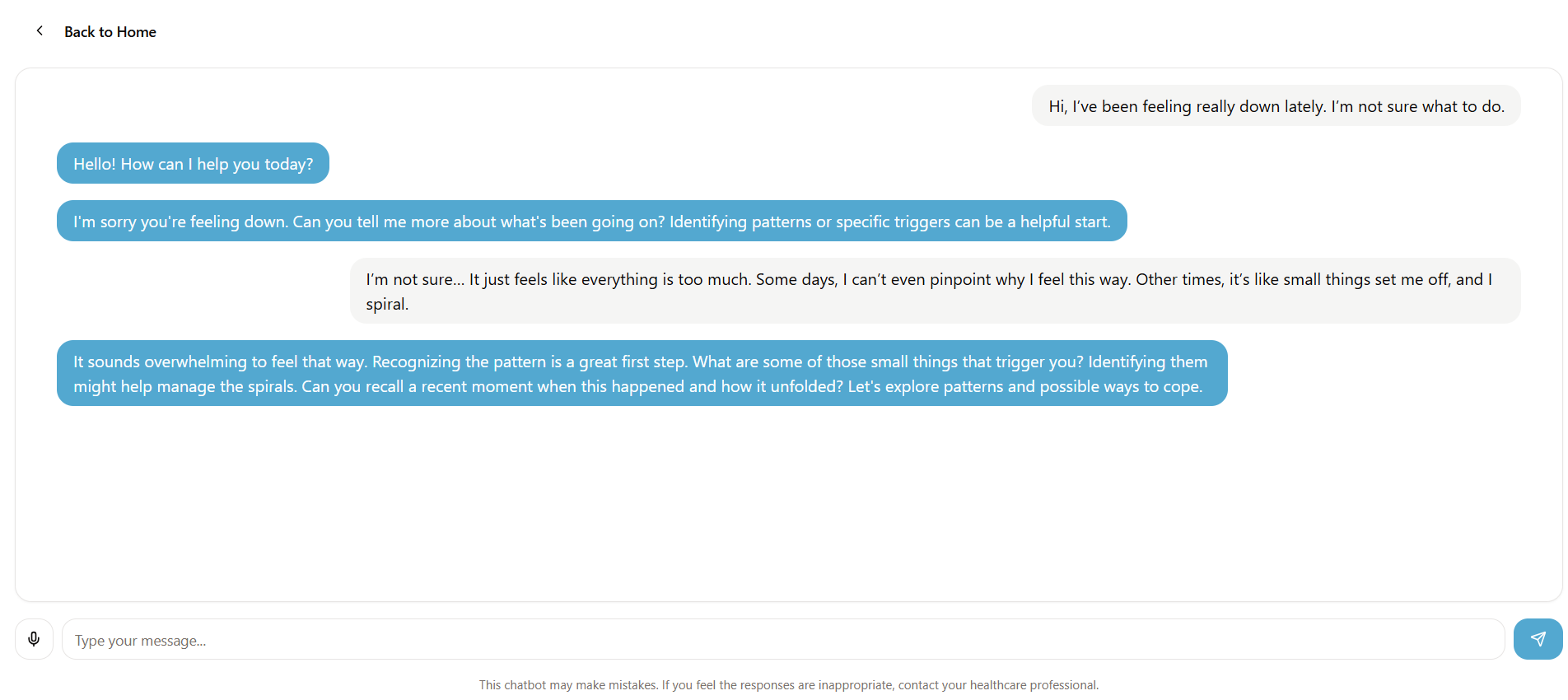


4. Psychological assessments


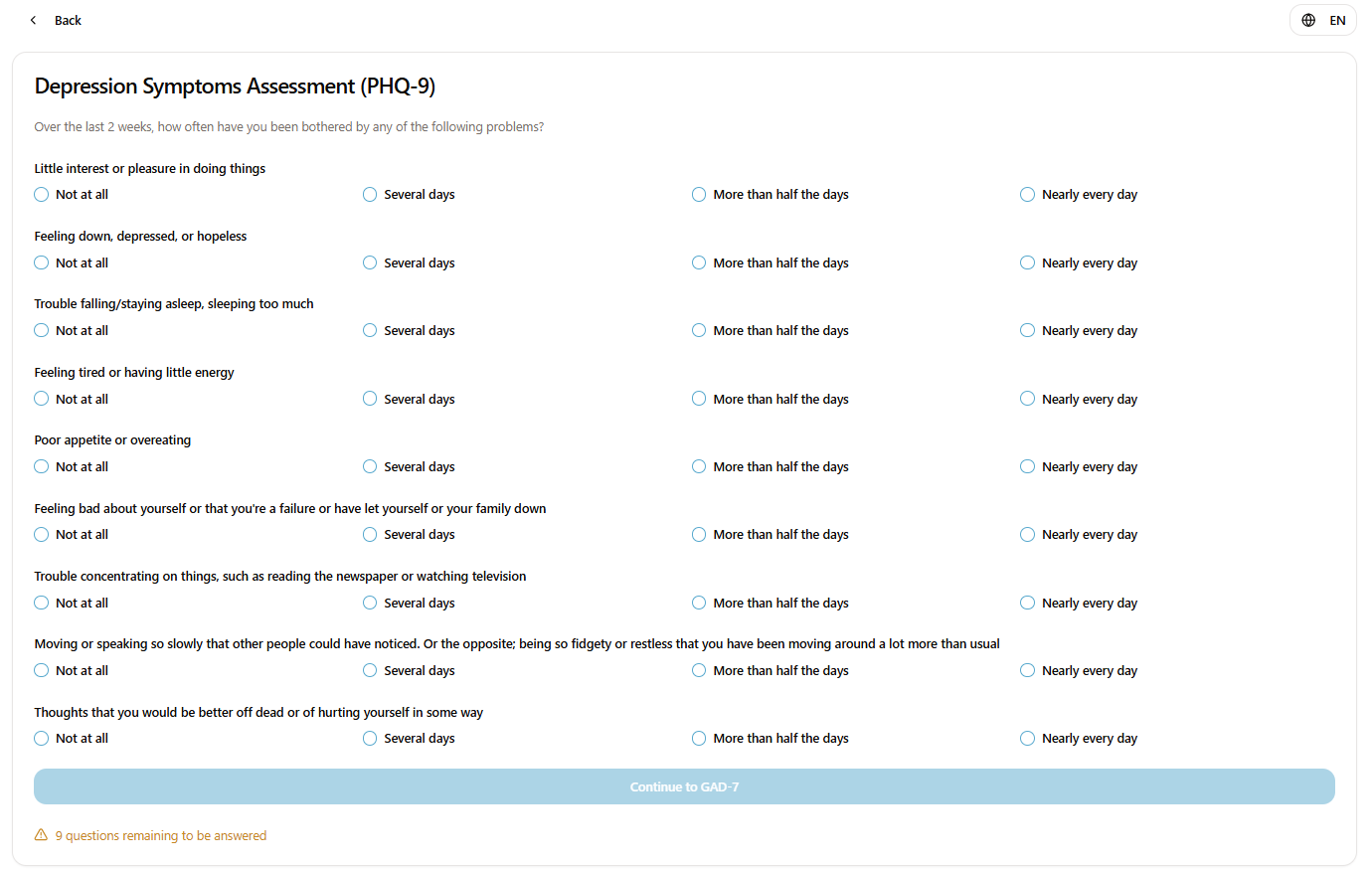


5. Therapeutic tasks.


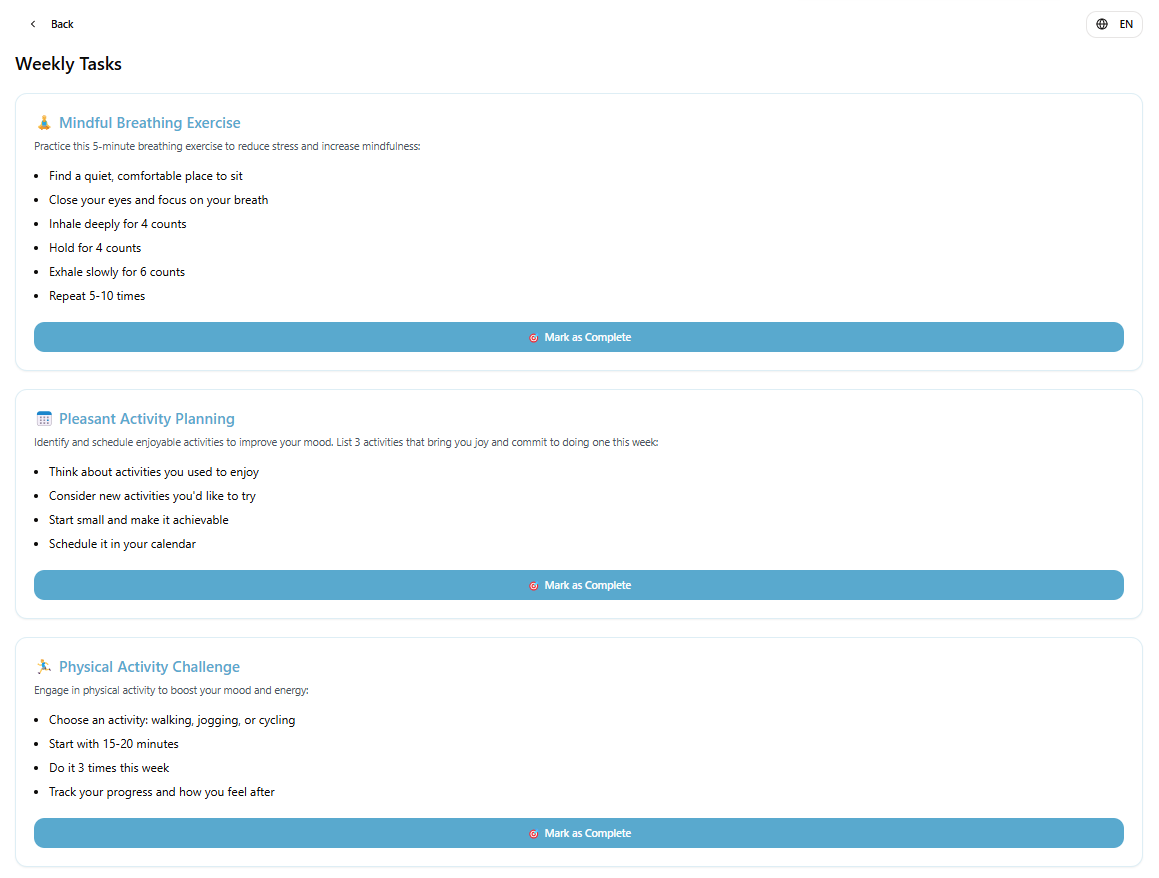

Supplement: S4 File — (DOCX) [file pone.0344939.s004.docx]
