# Supplementary material for: Development, system design, safety, and performance metrics of a conversational agent for reducing depressive and anxious symptoms based on a large language model: The MHAI study
Source: PLoS One. 2026 Mar 18;21(3):e0344939. doi: 10.1371/journal.pone.0344939 (PMC12998858; doi:10.1371/journal.pone.0344939)
Supplement: S1 Table — (DOCX) [file pone.0344939.s005.docx]

**S1 Table.** Technical characteristics for the frontend, backend, and database.

| Component | Description | Technologies / Tools |
| --- | --- | --- |
| Frontend | Users access the application via a web browser, where the user interface sends requests to the backend through HTTP calls. The retrieved data is dynamically presented in the interface. | React + TypeScript: A JavaScript framework with static typing.  Tailwind CSS: Used for responsive styling.  Shadcn/UI: A library of prebuilt UI components.  Wouter Router: Manages internal navigation within the application.  React Query: Optimizes state management and communication with the backend. |
| Backend | The backend acts as an intermediary between the frontend and the database, processing business logic and handling API requests to OpenAI. It receives HTTP requests from the frontend, processes data using the OpenAI API when needed, queries and updates the database through Drizzle ORM, and returns the processed information to the frontend. | Express.js + TypeScript: A lightweight and scalable framework for handling HTTP requests.  Drizzle ORM: Enables secure and efficient interaction with the PostgreSQL database.  OpenAI API: Processes AI requests for text generation.  MemoryStore: Temporarily manages in-memory sessions.  Nodemailer: Automates email delivery. |
| Database | A PostgreSQL database was used to store all application data. Retrieved information is sent back to the frontend. | The following tables and variables are used in the database:  users: {id, username, password, createdAt, lastLoginAt}  chatMessages: {id, userId, message, isBot, feedback, pageContext}  evaluations: {id, userId, type, score, answers}  taskCompletions: {id, userId, taskId, notes, weekStartDate}  userActivityLog: {id, userId, action, details, timestamp}  trainingDocuments: {id, title, content, fileType, uploadedBy} |
