# Supplementary material for: Development, system design, safety, and performance metrics of a conversational agent for reducing depressive and anxious symptoms based on a large language model: The MHAI study
Source: PLoS One. 2026 Mar 18;21(3):e0344939. doi: 10.1371/journal.pone.0344939 (PMC12998858; doi:10.1371/journal.pone.0344939)
Supplement: S2 Table — (DOCX) [file pone.0344939.s006.docx]

**S2 Table.** One-way sensitivity analysis.

| Intensity | Messages  per session | Cost per session  (LLaMA 3.1-8B) | Cost per session  (GPT-4o) | Total cost  (10 sessions)  (LLaMA 3.1-8B) | Total cost  (10 sessions)  (GPT-4o) |
| --- | --- | --- | --- | --- | --- |
| Low (1 msg/min) | 45 | $0.000174915 | $0.0220370625 | **$0.00174915** | **$0.220370625** |
| Moderate (2 msg/min) | 90 | $0.00034983 | $0.044074125 | **$0.0034983** | **$0.44074125** |
| High (3 msg/min) | 135 | $0.000524745 | $0.0661111875 | **$0.00524745** | **$0.661111875** |

Note: Cost in US dollars.
