# Supplementary figures and images for: Development, system design, safety, and performance metrics of a conversational agent for reducing depressive and anxious symptoms based on a large language model: The MHAI study
Source: PLoS One. 2026 Mar 18;21(3):e0344939. doi: 10.1371/journal.pone.0344939 (PMC12998858; doi:10.1371/journal.pone.0344939)

**S1 Fig.** Architecture and connection between the different technologies.
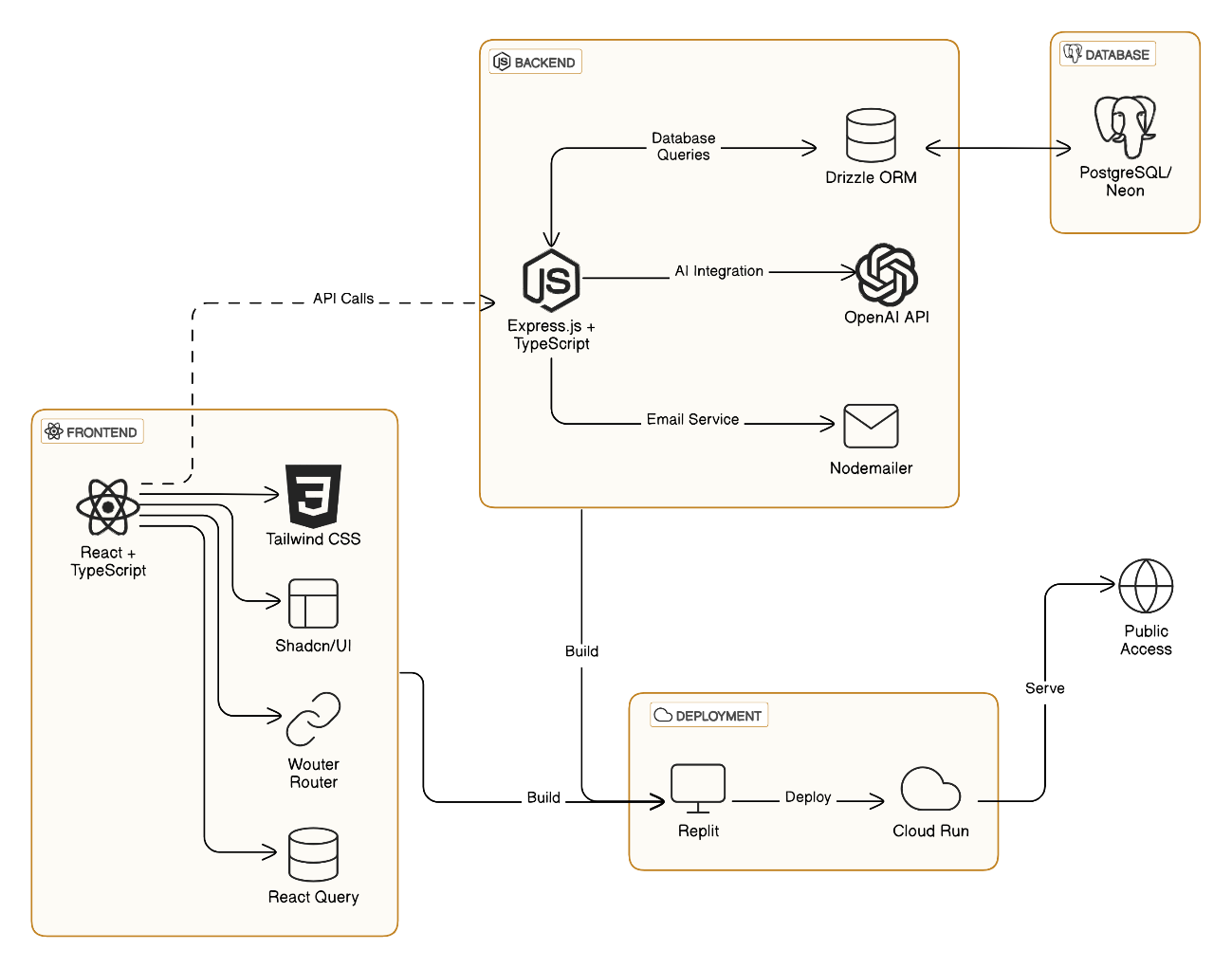

Supplement: S1 Fig — (DOCX) [file pone.0344939.s007.docx]
