# Supplementary material for: Development, system design, safety, and performance metrics of a conversational agent for reducing depressive and anxious symptoms based on a large language model: The MHAI study
Source: PLoS One. 2026 Mar 18;21(3):e0344939. doi: 10.1371/journal.pone.0344939 (PMC12998858; doi:10.1371/journal.pone.0344939)
Supplement: Attachment — Submitted filename: 3. Response letter.docx [file pone.0344939.s009.docx]

**PONE-D-25-55991**

**Development, System Design, Safety, and Performance Metrics of a Conversational Agent for Reducing Depressive and Anxious Symptoms Based on a Large Language Model: The MHAI Study**

Across reviewers, the manuscript is considered clearly written and supported by a well-articulated system architecture. However, significant strengthening is required before further consideration. Reviewer 1 requests the addition of an abbreviations table, clearer articulation of the manuscript structure and contributions, and an updated related work section with a synthesis table. Reviewer 2 highlights the need to address ecological validity (given reliance on simulated interactions), report inter-rater reliability, expand the analysis of cost–effectiveness trade-offs, correct timeline inconsistencies, and broaden the discussion of personas, safety incidents, and future extensions (e.g., fine-tuning or retrieval-augmented generation). Reviewer 3 raises concerns about the novelty, requesting a clearer articulation of the manuscript’s distinct scientific contribution, a more explicit positioning relative to closely related systems, and stronger empirical claims beyond descriptive comparisons.

**Reply:** Thank you very much for your review of the manuscript and for your constructive comments. We address each point below.

## Comments to the Author

### Reviewer #1:

This paper proposes a cross-sectional study in two phases. developed a mental health platform integrating a conversational agent with functionalities including personalized context, pretrained therapeutic modules, self-assessment tools, and an emergency alert system. Second, evaluated the agent’s responses in simulated interactions based on predefined user personas for each LLM. Four expert raters assessed 816 interaction pairs using a 5-criterion Likert scale evaluating tone, clarity, domain accuracy (correctness), robustness, completeness, boundaries, target language, and safety. In addition, we use performance metrics based on numerical criteria such as cost, response length, and number of tokens. Multiple linear regression models were used to compare LLM performance and assess metric interrelations.

First, developed a web-based mental health platform using a user-centered design, structured into frontend, backend, and database layers. The system integrates therapeutic chat (GPT-4o and Llama 3.1-8B), psychological assessments (PHQ-9, GAD89 7), CBT-based tasks, and an emergency alert system. The platform supports secure user authentication, data encryption, multilingual access, and session tracking.

Second, GPT4o outperformed Llama 3.1-8B in both performance metrics based on numerical criteria and Likert scale criteria, generating longer and more lexically diverse responses, using more tokens, and scoring higher in clarity, robustness, completeness, boundaries, and target language. However, it incurred higher costs, with no significant differences in tone, accuracy, or safety.

Good work keeps up
But some comments are needed?
submitted to editor

**Reply:** Thank you very much. We believe that the reviewer has a thorough understanding of the study.

Below, we respond to each of the comments.

### Reviewer #2:

This manuscript presents a methodologically rigorous investigation into the development and comparative evaluation of large language model (LLM)-based conversational agents for mental health interventions targeting depression and anxiety. The authors address a critical gap in the literature regarding transparency, reproducibility, and standardization in LLM evaluation for clinical applications. The study is structured in two phases: (1) system design and development documentation, and (2) controlled comparative evaluation of GPT-4o versus Llama 3.1-8B using standardized metrics.

Specific Strengths:

1. Methodological Transparency: The authors provide comprehensive technical documentation of the three-layer system architecture (frontend: React+TypeScript; backend: Express.js; database: PostgreSQL), addressing the critical issue that 99.3% of prior studies evaluated closed-source models without sufficient version identification. This level of transparency significantly enhances reproducibility.
2. Robust Statistical Framework: The sample size calculation is appropriately justified (minimum 788 interaction pairs for d=0.2, α=0.05, power=80%), and the final sample of 816 interaction pairs exceeds this threshold. The use of multiple linear regression models adjusted for language, user persona, and researcher effects demonstrates methodological sophistication.
3. Comprehensive Evaluation Framework: The study employs a dual assessment approach—(a) numerical performance metrics (response length, lexical diversity, token counts, cost analysis) and (b) eight Likert-scale criteria (tone, clarity, domain accuracy, robustness, completeness, boundaries, target language, safety)—which aligns with expert consensus recommendations from prior Delphi studies.
4. Open Science Practices: Full data availability via Figshare (DOI: 10.6084/m9.figshare.29606618.v1) and adherence to CHART reporting guidelines represent commendable scholarly practices.
5. Comparative Inclusion of Open-Source Models: The evaluation of Llama 3.1-8B alongside GPT-4o addresses concerns about over-reliance on proprietary systems and provides valuable insights for resource-constrained implementations.
**Reply:** Since all comments in this section focus on the positive aspects of the manuscript, we address them collectively. Thank you very much for your comments. We believe the second reviewer has correctly identified the manuscript's strongest points.

Major Methodological Concerns:

1. Ecological Validity Limitations: The exclusive reliance on simulated interactions conducted by four research team members, rather than authentic patient-agent exchanges, substantially limits the generalizability of findings to real-world clinical contexts. While the ethical justification for this approach is sound given potential risks from AI hallucinations, the absence of external validation with actual end-users represents a significant limitation.
Recommendation: The authors should explicitly acknowledge this limitation and discuss plans for phased real-world validation studies. Consider proposing a multi-stage validation framework: (a) expert evaluation (completed), (b) pilot testing with supervised participants, (c) randomized controlled trial.
**Reply:** We agree with the reviewer. We have modified the following limitation:

*“Second, the evaluation was conducted using research team members simulating user personas rather than real patient interactions. While this approach limits ecological validity, we considered it appropriate for initial safety evaluation to avoid potential ethical risks associated with exposing vulnerable populations to unvalidated systems. Therefore, in later phases of the study, the platform should be validated in real-world settings. For example, this includes studies with real users (patients) in supervised environments, followed by randomized clinical trials.”*

3. Inter-Rater Reliability Omission: Despite employing four independent raters to assess 816 interaction pairs using subjective Likert criteria, the manuscript does not report inter-rater reliability statistics (e.g., intraclass correlation coefficient, Fleiss' kappa). This omission is critical because subjective assessments of constructs like "tone," "clarity," and "robustness" require demonstrated consistency across evaluators to establish measurement validity.

Recommendation: Calculate and report inter-rater reliability coefficients for each Likert criterion. If reliability is suboptimal for certain dimensions, discuss implications for interpretation and propose refinements to assessment protocols.
**Reply:** In our study, the four evaluators did not rate the same interactions. Each researcher (evaluator) assessed a distinct subset of interaction pairs, generated from different conversations for each LLM. Therefore, due to the study design, it is not possible to validly estimate kappa or ICC across the entire dataset. However, to mitigate evaluator-related bias, we used mixed-effects models adjusted for evaluator, thereby preventing the main analysis from being affected by rater severity or leniency, that is, by an evaluator being systematically more stringent or more lenient.

We have added the following paragraph:

*“Fourth, because different evaluators rated different interactions, it was not possible to estimate inter-rater reliability coefficients (i.e., kappa or ICC). However, to mitigate evaluator-related bias, we used mixed-effects models adjusted for evaluator, thereby preventing the main analysis from being affected by rater severity or leniency, that is, by an evaluator being systematically more stringent or more lenient.”*

4. Cost-Effectiveness Analysis Depth: While the study reports that GPT-4o incurs approximately 140-fold higher costs than Llama 3.1-8B ($45.60 vs. $0.32 per 100K output tokens), there is insufficient discussion of the cost-performance trade-offs for implementation decisions. The clinical significance of GPT-4o's superior performance on clarity, robustness, completeness, boundaries, and target language metrics must be weighed against economic constraints in real-world healthcare settings.

Recommendation: Provide a dedicated subsection discussing cost-effectiveness from multiple stakeholder perspectives (public health systems, private clinics, low-resource settings). Consider threshold analyses: at what performance differential would the cost premium be justified?
**Reply:** It was not possible to conduct a cost-effectiveness study because clinical outcomes were not available. Instead, we performed a cost sensitivity analysis of a hypothetical care service, focusing only on token-based costs.

We added an additional limitation:

*“Sixth, a cost-effectiveness analysis or clinical benchmarking were not possible because, although information on the cost per input and output token was available, there was no information on the clinical effect of the intervention. Therefore, future studies, such as randomized clinical trials, should include cost-effectiveness evaluations of this type of digital mental health intervention.”*

In the method’s section, we added:

*“We conducted a one-way sensitivity analysis to contextualize the estimated token-based costs under realistic variations in session intensity. Using the mean prompt and completion token counts observed in our evaluation and the per-token prices. We computed the expected LLM execution cost for a standardized course of care comprising 10 sessions of approximately 45 minutes each. Assuming an interaction rate ranging from 1 to 3 model responses per minute (45–135 responses per session).”*

In the results section, we added:

“One-way sensitivity analysis of cost

The estimated total cost per patient by 10 sesions of 45 min each one, ranged from $0.22 to $0.66 for GPT-4o, compared with $0.0017 to $0.0053 for Llama 3.1-8B (see Supplementary Material 7). These results indicate that the relative cost difference is robust to plausible changes in message volume, although our estimates capture only variable token costs and exclude infrastructure and implementation overhead.”

In the discussion section, we added:

“Our study performs a sensitivity analysis based on the assumption of a potential mental health care service and considers token-based costs. Although this perspective is limited, as it does not account for implementation, supervision, data management, infrastructure, and other associated costs, it serves as a proxy that allows potential decision-makers and managers to identify a constant cost for packages of 10 sessions and for each individual session, depending on the model. It should be noted that the evidence suggests that, although the initial implementation process of digital mental health interventions is more costly than traditional care, these costs decrease when large volumes of care are considered [25,26].”

Minor Technical and Presentation Issues:

1. Temporal Inconsistencies (Lines 154-162): The Methods section states that the protocol received ethical approval on September 19, 2025, with data collection occurring September 20-21, 2025. However, Line 154-155 indicates GPT-4o was selected as "the most widely used OpenAI version at the time of the study (May 2025)". This five-month discrepancy requires clarification. Please verify and correct all study timeline references for internal consistency.
**Reply:** Thank you very much for the clarification. We have modified these sections to avoid ambiguity in the temporal sequence of the study. We added:

*“The performance of two LLMs was evaluated: GPT-4o, which was selected for being the most widely used OpenAI version at the time of writing the study protocol (May 2025), and Llama 3.1-8B, which was chosen for being an open-access, easy-to-implement, and free LLM.”*

*“The research protocol was written and planned in May 2025, was approved by the ethics committee on September 19, 2025, and the evaluation and recruitment took place between September 20 and 21, 2025.”*

2. Absence of Fine-Tuning and RAG: The authors explicitly note that neither fine-tuning nor Retrieval-Augmented Generation (RAG) was applied to either LLM (Lines 157-158). While this decision ensures comparability of base models, the Discussion should address how these techniques might alter comparative performance and whether future iterations will explore these enhancements.
**Reply:** We agree with the reviewer’s comment. Not using RAG could be considered a limitation of the study. Therefore, we have added the following paragraph:

*“Fifth, our study did not include fine-tuning or retrieval-augmented generation (RAG) at the model design stage. Although it has been shown that fine-tuned models exhibit performance comparable to general-purpose models for different health-related tasks [25], the use of RAG could represent an alternative to improve the model’s metric performance. Therefore, we estimate that subsequent stages of the study will include the use of RAG with transcripts of psychotherapy recordings, coding patient and therapist responses, and incorporating care manuals or clinical practice guidelines to improve the performance of the responses.”*

3. User Persona Documentation: While Supplementary Material 3 contains user persona specifications, the main manuscript provides minimal detail about persona construction methodology, clinical diversity, or alignment with actual patient profiles. Greater transparency regarding persona development would enhance reproducibility and allow readers to assess external validity.
**Reply:** We added:

*“Development of user personas and clinical rationale*

*The construction of user personas focused on the most frequent patient typologies observed in routine telehealth care at a specialized mental health center (Digital Health Centro Médico) and on the selection of clinically prioritized profiles commonly encountered in real-world practice at this center. The user personas were developed based on real cases that served as reference points and were intentionally selected to maximize clinical diversity across diagnoses and symptom severity levels. For each user persona, a set of clinical characteristics was specified, including diagnosis, severity, reason for seeking help, and patient context, as well as sociodemographic characteristics such as age, gender, occupation, and marital status.*

*To protect privacy and ensure anonymization, the personas were not direct reproductions of identifiable patients. Sociodemographic and contextual details were modified, combined, or generalized so that no persona corresponded to an identifiable individual. Names, dates, and unique events were not retained. This approach preserves clinical realism while minimizing the risk of reidentification. Supplementary Material 3 provides the complete specifications of the user personas.”*

4. Safety Incident Reporting: Although "safety" constitutes one of eight evaluation criteria (defined as absence of stigmatization, guilt induction, inappropriate medication recommendations, or self-harm encouragement), the Results section does not explicitly report whether any unsafe responses were identified. Table 3 shows no significant difference in safety scores between models (Llama 3.1-8B: 4.76±0.51 vs. GPT-4o: 4.77±0.46, p=0.844), but qualitative examples of edge cases or concerning responses would provide valuable context.
**Reply:** We added to the results section:

*“It should be noted that no interaction pairs that were clinically risky or unsafe received a score of 1, nor were there any particularly relevant cases that could be listed; therefore, no concrete examples of potentially unsafe interactions could be provided.”*

5. Limited Model Scope: The evaluation encompasses only two LLMs. While the inclusion of both proprietary and open-source models is valuable, the Discussion should acknowledge emerging alternatives (e.g., Claude 3, Gemini 2.0, DeepSeek, Mistral) and outline criteria for future comparative studies.
**Reply:** We added:

*“We encourage researchers to conduct future studies focused on developing technological solutions in mental health that can comply with existing regulations, such as the standards of the Health Insurance Portability and Accountability Act (HIPAA), or similar legislation in different countries that ensures data privacy and security [25]. Although commercial models such as Gemini, Claude, or GPT are at the frontier of current knowledge, they are not HIPAA-compliant, which limits their use in healthcare. Therefore, it is advisable to use open-source models, such as GPT-OSS:120B or DeepSeek-R1, provided that national legislation allows their use. Running models on local servers enables control over data and facilitates compliance with privacy and health information security laws.”*

*“First, we evaluated a limited number of LLMs, specifically GPT-4o and Llama 3.1-8B, without including other open-access models such as DeepSeek, larger Llama variants, or other state-of-the-art LLMs like Gemini 2.5-Pro, Grok-4, or GPT-o3. This limitation restricts both the generalizability of our findings and comparability with alternative models that may offer different performance-cost profiles.”*

Strongly recommended revisions:
4. Add cost-effectiveness analysis subsection with practical implementation guidance
**Reply:** Thank you very much for the recommendation; however, it was not possible to conduct a cost-effectiveness analysis due to the design of our study. We added an additional limitation:

*“Sixth, a cost-effectiveness analysis or clinical benchmarking were not possible because, although information on the cost per input and output token was available, there was no information on the clinical effect of the intervention. Therefore, future studies, such as randomized clinical trials, should include cost-effectiveness evaluations of this type of digital mental health intervention.”*

5. Provide qualitative examples of response differences between models
**Reply:** We consider this to be a highly relevant comment. However, our study already includes examples of how the different evaluated models interact, as shown in Table 4.

6. Discuss potential impact of fine-tuning and RAG on findings
**Reply:** We added:

*“Fifth, our study did not include fine-tuning or retrieval-augmented generation (RAG) at the model design stage. Although it has been shown that fine-tuned models exhibit performance comparable to general-purpose models for different health-related tasks [25], the use of RAG could represent an alternative to improve the model’s metric performance. Therefore, we estimate that subsequent stages of the study will include the use of RAG with transcripts of psychotherapy recordings, coding patient and therapist responses, and incorporating care manuals or clinical practice guidelines to improve the performance of the responses.”*

7. Enhance user persona methodology description
**Reply:** As noted in the other previous comment, the methodology used to design the user personas is described below:

*“Development of user personas and clinical rationale*

*The construction of user personas focused on the most frequent patient typologies observed in routine telehealth care at a specialized mental health center (Digital Health Centro Médico) and on the selection of clinically prioritized profiles commonly encountered in real-world practice at this center. The user personas were developed based on real cases that served as reference points and were intentionally selected to maximize clinical diversity across diagnoses and symptom severity levels. For each user persona, a set of clinical characteristics was specified, including diagnosis, severity, reason for seeking help, and patient context, as well as sociodemographic characteristics such as age, gender, occupation, and marital status.*

*To protect privacy and ensure anonymization, the personas were not direct reproductions of identifiable patients. Sociodemographic and contextual details were modified, combined, or generalized so that no persona corresponded to an identifiable individual. Names, dates, and unique events were not retained. This approach preserves clinical realism while minimizing the risk of reidentification. Supplementary Material 3 provides the complete specifications of the user personas.”*

Optional enhancements:
8. Include sensitivity analyses examining whether results vary by user persona or language
**Reply:** We apologize for not being able to perform this analysis, as there was not a sufficient sample size to replicate the main analysis stratified by each user persona.

*“Seventh, it was not possible to perform a sensitivity analysis of the findings for each LLM and user persona, as the available sample size was insufficient to replicate the main analysis stratified by these different conditions.”*

9. Propose concrete validation roadmap for progression to real-world testing
**Reply:** We consider that this recommendation is beyond the central objective of the study.

Conclusion:
This manuscript makes substantive contributions to the methodological standardization of LLM evaluation in mental health applications. The technical development is sound, the statistical analyses are appropriate, and the comparative framework is valuable for the field. The identified concerns are addressable through targeted revisions that will strengthen the manuscript's impact and clinical applicability. With minor revisions, this work will constitute a significant addition to the digital mental health literature.
**Reply:** Thank you very much for your comments. We have addressed each of your queries in the section above of the manuscript.

Recommendation: Minor Revision

### Reviewer #3:

Dear Editor and Authors,
________________________________________
Summary of the Study
The study evaluates the design, safety framework, and performance of a large language model (LLM)-based conversational agent intended to support individuals with depressive and anxiety symptoms. The manuscript primarily focuses on system-level integration and a comparative evaluation of two existing language models (GPT-4o and Llama 3.1-8B) using simulated conversational scenarios assessed by expert reviewers. The evaluation relies on predefined quantitative metrics and Likert-scale-based qualitative judgments. While the topic is timely and relevant, the study does not clearly demonstrate a novel system development or a distinct scientific contribution beyond descriptive comparison.

________________________________________
Strengths of the Study
• The manuscript addresses a relevant and contemporary topic in digital mental health and AI-assisted interventions.
• The system architecture and safety considerations are described in a clear and organized manner.
• The authors employ a structured evaluation framework and follow established reporting guidelines.
• The comparative analysis between a proprietary and an open-source LLM is practically informative.
**Reply:** As the comments focus on the strengths of our study, we provide a joint response to all of them. Thank you very much for your review; we consider that the reviewer fully understands the content of our manuscript. We agree that the points highlighted as strengths are accurate.

________________________________________
Weaknesses of the Study
• The manuscript does not clearly articulate a novel scientific or methodological contribution.
**Reply:** We agree that the scientific contribution was not clearly articulated in the previous version of the manuscript. We have now incorporated a dedicated paragraph in the Introduction section to clarify this contribution.

*“The main scientific contribution of this study to the field of digital mental health is the proposal of a reproducible comparative evaluation framework that integrates standardized metrics of conversational quality, safety, and performance, applied systematically to an open-source language model and a commercial (closed-source) language model under controlled conditions. Unlike previous evaluations, the analysis is based on clinically plausible simulated interactions, adjusted for language, user persona, and evaluator, and simultaneously incorporates linguistic indicators, validated qualitative criteria, and economic metrics. In addition, the study presents a functional and integrated architecture of an LLM-based mental health platform, designed for early safety evaluations and future clinical studies, providing a replicable framework for subsequent research and for the responsible implementation of these systems in mental health care settings. Therefore, we have two study objectives: (1) to develop an LLM-based conversational agent through system design analysis and initial functionality testing, and (2) to evaluate its safety and performance through standardized assessment in controlled simulated interactions focused on depression and anxiety.”*

• The work relies exclusively on existing language models without introducing new algorithms, learning strategies, or adaptive mechanisms.
**Reply:**We agree with the reviewer that our study does not introduce a new algorithm or a specific new learning mechanism. However, we consider that the scientific value of this work does not lie in proposing a new LLM, but in demonstrating the technical and methodological feasibility of integrating existing models into a digital mental health platform and evaluating them comparatively under a standardized framework of conversational performance, safety, and cost, under controlled conditions.

At this stage, we decided not to apply fine-tuning or Retrieval-Augmented Generation (RAG) for a methodological reason: we aimed to estimate the performance and safety “floor” of the base models, avoiding improvements through additional engineering that could obscure fundamental differences between architectures or confound the interpretation of the findings. We included the following as a limitation:

*“Fifth, our study did not include fine-tuning or retrieval-augmented generation (RAG) at the model design stage. Although it has been shown that fine-tuned models exhibit performance comparable to general-purpose models for different health-related tasks [26], the use of RAG could represent an alternative to improve the model’s metric performance. Therefore, we estimate that subsequent stages of the study will include the use of RAG with transcripts of psychotherapy recordings, coding patient and therapist responses, and incorporating care manuals or clinical practice guidelines to improve the performance of the responses.”*

• Evaluation is limited to simulated interactions and expert ratings, with no real user involvement.
**Reply:** We agree with the reviewer, and we have added the following paragraph:

*“Second, the evaluation was conducted using research team members simulating user personas rather than real patient interactions. While this approach limits ecological validity, we considered it appropriate for initial safety evaluation to avoid potential ethical risks associated with exposing vulnerable populations to unvalidated systems. Therefore, in later phases of the study, the platform should be validated in real-world settings. For example, this includes studies with real users (patients) in supervised environments, followed by randomized clinical trials.”*

• The study reads largely as a system description and comparison rather than hypothesis-driven research.
**Reply:** We have revised the structure of our manuscript to focus on a hypothesis-driven evaluation. We highlight several paragraphs that have been modified throughout the manuscript:

*“Moreover, based on the second objective, we hypothesize that in the simulated interactions GPT-4o will obtain higher scores than Llama 3.1-8B on the performance metrics and safety.”*

*“For the main hypothesis (second objective), we evaluated which of the LLMs demonstrated better performance metrics and safety based on multiple linear regression models. Model adjusted by language (Spanish/English), user persona, and researcher. In addition, some of the responses were presented descriptively as examples of the style of responses for each model. We considered the hypothesis to be supported when the differences in performance metrics and safety were statistically significant (p < 0.05) and favored GPT-4o.”*

*“Our study evaluated the performance metrics based on quantitative response characteristics of two LLMs (GPT-4o vs. Llama 3.1-8B) and found that GPT-4o produced longer responses, with an average of 71.59 more characters than Llama 3.1-8B (95% CI: 51.23 to 91.94; p < 0.001), and exhibited greater lexical diversity, with a 7.77% increase compared to Llama 3.1-8B (95% CI: 6.86 to 8.69; p < 0.001). Additionally, GPT-4o used, on average, 3.16 more tokens for the input (95% CI: 1.37 to 4.95; p < 0.001) and 26.86 more tokens for the output (95% CI: 22.77 to 30.94; p < 0.001) than Llama 3.1-8B. This also translated into a higher cost per 100,000 tokens in USD for GPT-4o compared to Llama 3.1-8B (see Table 3).*

*In terms of conversational quality performance metrics, GPT-4o outperformed Llama 3.1-8B in several domains such like clarity of responses, the ability to handle ambiguous or incomplete questions while maintaining conversational coherence (robustness), comprehensive answers without including unsolicited information (completeness), the ability to restrict responses to mental health related content (boundaries), and the ability to respond in the same language in which the question was asked (target language) (see Table 3). No significant differences were observed in tone, domain accuracy, or response safety.*

*Consistent with our hypothesis, GPT-4o scored higher than Llama 3.1–8B on clarity, robustness, completeness, boundaries, and target language, while no differences were observed for tone, domain accuracy, or safety.”*

*“Our findings partially support our hypothesis, as GPT-4o outperformed Llama 3.1-8B only on selected conversational quality metrics. Specifically, GPT-4o demonstrated statistically superior performance in completeness, clarity, robustness, boundary adherence, and target language conformity. This difference may be attributed to the substantially smaller model size and lower parameter count of Llama 3.1-8B compared to GPT-4o’s higher capacity, which enables it to address complex benchmarks such as Humanity’s Last Exam, a level of performance that Llama 3.1-8B does not achieve.”*

________________________________________
Discussion of Specific Areas for Improvement
Major Issues
1. The manuscript does not explicitly define its unique contribution to the literature. It is unclear whether the novelty lies in system design, evaluation methodology, or safety implementation, as all major components rely on established practices.

4. The manuscript lacks a focused comparison with closely related studies to clarify how this work advances beyond previously published systems and evaluations.
*We repeat the comment provided above, as it refers to the same question raised by the reviewer.*

**Reply:** We agree that the scientific contribution was not clearly articulated in the previous version of the manuscript. We have now incorporated a dedicated paragraph in the Introduction section to clarify this contribution.

*“The main scientific contribution of this study to the field of digital mental health is the proposal of a reproducible comparative evaluation framework that integrates standardized metrics of conversational quality, safety, and performance, applied systematically to an open-source language model and a commercial (closed-source) language model under controlled conditions. Unlike previous evaluations, the analysis is based on clinically plausible simulated interactions, adjusted for language, user persona, and evaluator, and simultaneously incorporates linguistic indicators, validated qualitative criteria, and economic metrics. In addition, the study presents a functional and integrated architecture of an LLM-based mental health platform, designed for early safety evaluations and future clinical studies, providing a replicable framework for subsequent research and for the responsible implementation of these systems in mental health care settings. Therefore, we have two study objectives: (1) to develop an LLM-based conversational agent through system design analysis and initial functionality testing, and (2) to evaluate its safety and performance through standardized assessment in controlled simulated interactions focused on depression and anxiety.”*

2. Despite claims of system development, the study does not introduce a new model, algorithm, or adaptive framework. The work is primarily an integration and comparison of pre-existing LLMs.
*We repeat the comment provided above, as it refers to the same question raised by the reviewer.*

**Reply:**We agree with the reviewer that our study does not introduce a new algorithm or a specific new learning mechanism. However, we consider that the scientific value of this work does not lie in proposing a new LLM, but in demonstrating the technical and methodological feasibility of integrating existing models into a digital mental health platform and evaluating them comparatively under a standardized framework of conversational performance, safety, and cost, under controlled conditions.

At this stage, we decided not to apply fine-tuning or Retrieval-Augmented Generation (RAG) for a methodological reason: we aimed to estimate the performance and safety “floor” of the base models, avoiding improvements through additional engineering that could obscure fundamental differences between architectures or confound the interpretation of the findings. We included the following as a limitation:

*“Fifth, our study did not include fine-tuning or retrieval-augmented generation (RAG) at the model design stage. Although it has been shown that fine-tuned models exhibit performance comparable to general-purpose models for different health-related tasks [26], the use of RAG could represent an alternative to improve the model’s metric performance. Therefore, we estimate that subsequent stages of the study will include the use of RAG with transcripts of psychotherapy recordings, coding patient and therapist responses, and incorporating care manuals or clinical practice guidelines to improve the performance of the responses.”*

3. The exclusive use of simulated conversations and expert evaluations limits the ecological validity and weakens the empirical strength of the conclusions.
*We repeat the comment provided above, as it refers to the same question raised by the reviewer.*

**Reply:** We agree with the reviewer, and we have added the following paragraph:

*“Second, the evaluation was conducted using research team members simulating user personas rather than real patient interactions. While this approach limits ecological validity, we considered it appropriate for initial safety evaluation to avoid potential ethical risks associated with exposing vulnerable populations to unvalidated systems. Therefore, in later phases of the study, the platform should be validated in real-world settings. For example, this includes studies with real users (patients) in supervised environments, followed by randomized clinical trials.”*

________________________________________
Minor Issues
1. Some tables and figures could be reduced or moved to supplementary materials to improve readability.
**Reply:** We agree with the reviewer, and we have reduced the main manuscript to two figures and three tables.

2. Minor grammatical and stylistic issues are present, particularly in the Discussion section.
**Reply:** Reply: We have thoroughly revised the entire manuscript and implemented additional changes to improve readability and clarity, with particular attention to the Discussion section.

3. The manuscript does not report inter-rater reliability metrics for expert assessments.
**Reply:** We added:

*“Fourth, because different evaluators rated different interactions, it was not possible to estimate inter-rater reliability coefficients (i.e., kappa or ICC). However, to mitigate evaluator-related bias, we used mixed-effects models adjusted for evaluator, thereby preventing the main analysis from being affected by rater severity or leniency, that is, by an evaluator being systematically more stringent or more lenient.”*

4. The cost analysis lacks sensitivity analysis and contextual benchmarking.

**Reply:** In the method’s section, we added:

*“We conducted a one-way sensitivity analysis to contextualize the estimated token-based costs under realistic variations in session intensity. Using the mean prompt and completion token counts observed in our evaluation and the per-token prices. We computed the expected LLM execution cost for a standardized course of care comprising 10 sessions of approximately 45 minutes each. Assuming an interaction rate ranging from 1 to 3 model responses per minute (45–135 responses per session).”*

In the results’s section, we added:

“One-way sensitivity analysis of cost

The estimated total cost per patient by 10 sesions of 45 min each one, ranged from $0.22 to $0.66 for GPT-4o, compared with $0.0017 to $0.0053 for Llama 3.1-8B (see Supplementary Material 7). These results indicate that the relative cost difference is robust to plausible changes in message volume, although our estimates capture only variable token costs and exclude infrastructure and implementation overhead.”

We added an additional limitation:

*“Sixth, a cost-effectiveness analysis or clinical benchmarking were not possible because, although information on the cost per input and output token was available, there was no information on the clinical effect of the intervention. Therefore, future studies, such as randomized clinical trials, should include cost-effectiveness evaluations of this type of digital mental health intervention.”*

In the discussion section, we added:

“Our study performs a sensitivity analysis based on the assumption of a potential mental health care service and considers token-based costs. Although this perspective is limited, as it does not account for implementation, supervision, data management, infrastructure, and other associated costs, it serves as a proxy that allows potential decision-makers and managers to identify a constant cost for packages of 10 sessions and for each individual session, depending on the model. It should be noted that the evidence suggests that, although the initial implementation process of digital mental health interventions is more costly than traditional care, these costs decrease when large volumes of care are considered [25,26].”
