# Supplementary material for: Development, system design, safety, and performance metrics of a conversational agent for reducing depressive and anxious symptoms based on a large language model: The MHAI study
Source: PLoS One. 2026 Mar 18;21(3):e0344939. doi: 10.1371/journal.pone.0344939 (PMC12998858; doi:10.1371/journal.pone.0344939)
Supplement: Attachment — Submitted filename: 3._Response_letter_auresp_2.docx [file pone.0344939.s010.docx]

PONE-D-25-55991R1

Development, System Design, Safety, and Performance Metrics of a Conversational Agent for Reducing Depressive and Anxious Symptoms Based on a Large Language Model: The MHAI Study

**Review Comments to the Author**

Reviewer #1: This study presents a conversational agent with multiple functionalities and shows that GPT-4o outperforms Llama 3.1-8B in performance, although at a higher cost. This platform could be used in future clinical trials or real-world implementation studies.
Good work keep up

**Reply:** Thank you very much for your comments.

Reviewer #2: The authors have substantially addressed the major concerns raised in the previous review round. The manuscript demonstrates methodological rigor, transparent reporting, and adherence to CHART guidelines. The comparative evaluation of GPT-4o versus Llama 3.1-8B provides valuable insights into LLM performance for mental health applications.
Strengths of the Revision:
1. Comprehensive Response to Reviewers: All three reviewers' comments have been thoroughly addressed with detailed explanations and appropriate manuscript modifications.
2. Enhanced Methodological Transparency: The addition of seven new limitations strengthens the manuscript's scientific integrity, including acknowledgment of ecological validity constraints, inter-rater reliability limitations, absence of cost-effectiveness analysis, and lack of fine-tuning/RAG implementation.
3. Scientific Contribution Clarification: The newly added paragraph in the Introduction clearly articulates the study's unique contribution: a reproducible comparative evaluation framework integrating standardized conversational quality, safety, and performance metrics under controlled conditions.
4. Improved User Persona Documentation: The detailed methodology for persona development (based on real clinical cases with anonymization protocols) significantly enhances reproducibility.
5. Addition of Sensitivity Analysis: The one-way sensitivity analysis for cost estimation (10 sessions × 45 minutes scenarios) provides practical implementation context.
6. Statistical Rigor: The use of mixed-effects models adjusted for evaluator, language, and user persona effectively mitigates potential biases.
**Reply:** We address the six comments collectively, as they focus on the strengths of our study. We thank the reviewer for the comments provided in the previous round; they have helped us strengthen the manuscript and improve its scientific rigor.

Remaining Limitations:
1. Ecological Validity: While the authors appropriately justify the use of simulated interactions for initial safety evaluation, the manuscript would benefit from a more concrete roadmap for real-world validation phases (e.g., timeline for pilot testing with supervised participants).
**Reply:** We have added the following to the subsection “Implications for future studies”:
*“Our study outlines a pathway for future research that addresses the limitations identified and ensures a substantial contribution to the field of digital mental health. First, we require evaluations with real users in controlled settings, that is, a preliminary usability assessment with patients and therapists to ensure that the platform is acceptable, usable, and can be adopted as a complement to psychotherapeutic processes. In addition, we should assess additional requirements that may be valuable to users and were not previously considered. Second, to ensure the ecological validity of the findings in real-world contexts, we must conduct a preliminary effectiveness evaluation in small groups, such as pilot clinical trials, or by using non-experimental methods, such as case series without a control group. This would allow us to determine whether the observed results are replicable in routine care settings and to assess preliminary clinical efficacy. Moreover, it would help identify the optimal platform prescription, such as the frequency of use and the number of sessions.”*

2. Clinical Effectiveness Gap: The absence of clinical outcome measures limits the ability to perform cost-effectiveness analysis. Consider elaborating on plans to incorporate validated clinical endpoints (e.g., PHQ-9/GAD-7 pre-post changes) in future trials.
**Reply:** We have added the following to the subsection “Implications for future studies”:

*“Second, to ensure the ecological validity of the findings in real-world contexts, we must conduct a preliminary effectiveness evaluation in small groups, such as pilot clinical trials, or by using non-experimental methods, such as case series without a control group. This would allow us to determine whether the observed results are replicable in routine care settings and to assess preliminary clinical efficacy. Moreover, it would help identify the optimal platform prescription, such as the frequency of use and the number of sessions. Third, we should conduct robust clinical studies with control groups and adequate sample sizes. This would allow us to establish clinical efficacy under controlled conditions.”*

3. Generalizability to Other LLMs: The evaluation is limited to GPT-4o and Llama 3.1-8B. While the authors acknowledge this limitation, explicitly discussing selection criteria for future model comparisons would strengthen the framework's applicability.
**Reply:** Added to the limitations subsection:

*“First, we evaluated only two LLMs, GPT-4o and Llama 3.1-8B, without including other open-access models such as DeepSeek, larger Llama variants, or other state-of-the-art models such as Gemini 2.5-Pro, Grok-4, or GPT-o3. This restricts the generalizability of our findings and limits comparison with models that may present different performance–cost profiles. Although our results cannot be directly generalized to other LLMs, prior evidence indicates that state-of-the-art reasoning models generally outperform older or non-reasoning models [28]. Therefore, while our findings may not extend to earlier models, it is plausible that more recent state-of-the-art models would demonstrate similar or superior performance.”*

Minor Recommendations:
- Consider adding a brief statement on platform scalability and infrastructure requirements for real-world deployment
**Reply:** We have added the following to the subsection “Implications for future studies”:

*“At the architectural level of MHAI, the platform must implement additional servers to support high traffic volumes through horizontal scalability, while maintaining compliance with data security and privacy standards such as HIPAA. Moreover, MHAI should not operate as an isolated platform; instead, it should be integrated into a real clinical care process and interoperable with electronic health record systems such as OpenMRS, Epic, or Cerner.*

*Regarding new features and functions, several developments are required. First, the emergency button must be tested under real-world conditions with real users, as only patients can determine whether it is helpful in practice. Although the current implementation is functional and operates correctly, it has not yet been tested in real-world contexts due to ethical constraints. Second, functions that allow users to obtain information from a licensed health professional, schedule a new appointment, or be redirected to a real clinical care pathway should be implemented. Third, additional large language models should be implemented to review conversations and extract clinically relevant information that can be validated by a health professional and subsequently entered into the patient’s electronic health record. For example, one model could identify suicide risk in conversations, while another could detect signs and symptoms that can later be validated and documented in the medical record.”*

- Clarify whether the emergency alert system has been tested in pilot scenarios
**Reply:** We have added the following to the subsection “Implications for future studies”:

*“Regarding new features and functions, several developments are required. First, the emergency button must be tested under real-world conditions with real users, as only patients can determine whether it is helpful in practice. Although the current implementation is functional and operates correctly, it has not yet been tested in real-world contexts due to ethical constraints.”*

- Discuss potential regulatory considerations (e.g., FDA guidance on clinical decision support software, if applicable to your jurisdiction)

**Reply:** We have added the following to the subsection “Implications for future studies”:

*“Fourth, we should perform implementation studies in primary care and hospital settings to evaluate the real-world impact of MHAI in clinical contexts. At the same time, registration with the relevant regulatory authorities, such as the Food and Drug Administration (FDA) in the United States or the Dirección General de Medicamentos, Insumos y Drogas (DIGEMID) in Peru, will be required if the platform is to be considered a medical device for therapeutic use. Prior to this stage, the platform should be regarded as a tool that may improve overall emotional well-being, rather than as a formal therapy.”*
